# An intervention study on a hospital-community integrated management model of tobacco dependence based on a community intervention trial

**DOI:** 10.3389/fpsyt.2023.1029640

**Published:** 2023-03-07

**Authors:** Kun Qiao, Han Liu, Xingming Li, Qianying Jin, Yao Wang, Mingyu Gu, Xinyuan Bai, Tingting Qin, Yutong Yang

**Affiliations:** ^1^School of Public Health, Capital Medical University, Beijing, China; ^2^Beijing Friendship Hospital, Capital Medical University, Beijing, China; ^3^Sir Run Run Shaw Hospital, School of Medicine, Zhejiang University, Hangzhou, Zhejiang, China

**Keywords:** smoking cessation, community intervention trial, smoking cessation clinic, community health center, integrated intervention

## Abstract

**Objective:**

To assess the effect of the hospital-community integrated management model of tobacco dependence on smoking cessation among community residents compared with a brief smoking cessation intervention.

**Methods:**

Our study recruited 651 smokers who were willing to quit in 19 communities in Beijing and conducted a 6-month smoking cessation intervention. The control group receiving a brief smoking cessation intervention and the pilot group receiving an integrated smoking cessation intervention. Intention-to-treat analysis (ITT) and generalized estimating equations were used to assess the effects of the integrated intervention and smoking cessation medication on average number of cigarettes smoked per day (ACSD) and smoking cessation rate.

**Results:**

Simple effects analysis showed that smokers taking medication had significantly lower ACSD than those not taking medication at follow-up, the control group reduced smoking by 3.270, 4.830, and 4.760 cigarettes in the first, third and sixth months, respectively, while the pilot group reduced by 6.230, 5.820, and 4.100 cigarettes. The integrated intervention significantly reduced ACSD among medication-taking smokers at 1st month (reduced by 3.420, *P* < 0.05) and 3rd month (reduced by 2.050, *P* < 0.05), but had no significant effect among non-medication taking smokers. The 3rd month smoking cessation rate among medication-taking smokers was 27.0%, which was significantly higher than the smokers with brief smoking cessation intervention.

**Conclusion:**

The integrated hospital-community intervention can significantly promote smoking cessation among smokers taking medication, but the issue of payment for medication and additional labor compensation for medical staff should be addressed before its popularization.

## 1. Introduction

Tobacco harm is a significant public health problem in the world today. Smoking can lead to cardiac disease, chronic respiratory disease, cancer, and many other diseases (1). In 2019, more than 8 million people died from tobacco-related diseases worldwide (2). China is the world’s largest producer and consumer of tobacco, with nearly 300 million smokers. In China, the smoking rate among people aged 15 years and older was 23.5% in 2020, far above the global average of 17.0% (2). In addition, more than 1 million people die each year from smoking-related diseases in China, which will increase to 2 million per year by 2030 if no effective action is taken (3). Smoking cessation is the top priority for preventing smoking-related diseases (4). The earlier smokers quit smoking and the longer they continue, the greater health gains they will achieve. Despite this, the willingness of Chinese smokers to quit is low (5). According to the Report of International Tobacco Control (ITC) Policy Evaluation Project China Survey Round 1–5, the proportion of smokers in China who have no intention to quit is as high as 59%. Another survey showed that 19.8% of those who had smoked in the past 12 months had tried to quit, 16.1% of current smokers intended to quit within the next 12 months, and only 5.6% of smokers planned to quit within 1 month (6).

Scientific and professional guidance is indispensable to quit smoking. Smokers’ success in quitting is influenced by both individual factors, such as their own health status and economic status, and social factors, such as family environment, interpersonal, relationships and policies. The main smoking cessation interventions commonly used internationally are psychological and behavioral interventions, pharmacological therapies, telephone intervention therapies, and integrated interventions, etc. (7). Smoking cessation clinics are an effective way to implement smoking cessation interventions, and they are irreplaceable in persuading smokers to quit. Some countries and institutions have also started to implement smoking cessation clinics on a large scale, with considerable success (8). However, in China, the rate of independent smoking cessation among residents is high. The ITC survey showed that the proportion of tobacco-dependent patients in China who did not use any method in quitting smoking was as high as 90.1%. In a study by Yang et al. (9), 87.6% of smokers had not received help to quit. In addition, although smoking cessation clinics are well equipped with hardware and other aspects, the consultation rate of smoking cessation clinics in China has been very low for a long time (8, 10). The main reasons for this are the low awareness of smoking cessation clinics, the self-payment for smoking cessation medications, the lack of specific drugs for medications and adverse drug reactions, etc. (11).

Tobacco dependence is a chronic disease with a long-term treatment. Community-based smoking cessation interventions can provide comprehensive, continuous, and proactive management for smokers. The main focus of community-based smoking cessation interventions abroad is not limited to community health centers. A study in the United States used external resources such as the media and social organizations to create a social climate that did not support smoking behavior to increase smoking cessation rate (12). In the UK, pharmacists or pharmacy assistants in community pharmacies provide smokers medication counseling, smoking cessation education, and health behavior advice, combined with smoking cessation medications for intervention (13). A study in Thailand combined pharmacists and community health workers (CHWs) to deliver smoking cessation interventions to smokers (14). Compared to smoking cessation clinics in general hospitals, communities have advantages in terms of geography and price. In China, cessation interventions in community clinics mainly consist of providing health education on the dangers of tobacco and brief smoking cessation interventions (15, 16). The main problem is the lack of long-term effective systematic management, continuous technical support and social support environment for smoking cessation. In conclusion, the integrated hospital-community model has become another feasible way to solve the current problem of smoking cessation in China.

Based on the above status, the program team designed a study to optimize the hospital and community-based tobacco dependence management model by focusing on hospital and community strengths. This paper uses adult smokers who participated in the program as the study population to evaluate the effectiveness of this integrated intervention on smoking cessation for community residents in Beijing.

## 2. Materials and methods

### 2.1. Design

Search for Optimization of Tobacco Dependence Management Model Based on Hospital and Community (2017YFC1309404) was funded by the 2017 Key Research and Development Program of the Ministry of Science and Technology of China. This is a community intervention trial implemented in Beijing, with 19 community health service centers selected as investigation and follow-up sites in Beijing by convenience sampling method from December 2019 to June 2020.

### 2.2. Participants

Before the implementation of the intervention, this study conducted program publicity in community health centers to recruit smokers who were willing to quit. The general practitioner team helped to mobilize smokers to attend and encourage them to participate in the program. Sample size calculation was based on the 2018 National Adult Tobacco Survey report (6), which showed that the proportion of smokers who tried to quit within the past 12 months was 19.8%, the 95% CI corresponded to a Z value of 1.96 and the accuracy δ value was taken as 0.05. And the sample size calculation formula used was:


(1) $$n=\frac{Z^{2}*\left(\pi *\left(1-\pi \right)\right)}{\delta^{2}}$$

$$=\frac{1.96^{2}*\left(0.198*\left(1-0.198\right)\right)}{0.05^{2}}=245$$

The minimum sample size required was calculated to be 245 each group. In this study, 683 willing quitters were finally recruited by screening according to the following inclusion and exclusion criteria.

Inclusion criteria: ➀ Age 18 years or older; ➁ Smokers who are permanent residents of the community and can complete subsequent follow-up; ➂ Patients who can communicate fluently and are willing to participate in; ➃ Willingness to quit smoking; and ➄ Signing an informed consent form.

Exclusion criteria: ➀ Below age 18; ➁ Non-smokers; ➂Those with serious mental illness or unable to communicate; and ➃ Smokers who have no intention to quit at all or are unwilling to participate in the trial.

At the baseline, smokers who met the inclusion and exclusion criteria had one-to-one questionnaires completed by the investigators. Before the intervention, investigators received unified and systematic training. The training included familiarization with the questionnaire structure, explanation of questionnaire terminology, common problems in questionnaire completion, and the use of the CO monitor (Micro+ Smokerlyzer, produced by Bedfont). After completing the questionnaire, the investigators measured the CO levels of the respondents through the CO monitor and recorded them on the questionnaire. Finally, if the survey respondents’ community was included as the pilot group community, the respondents were added to the WeChat group chat of that community. WeChat is the most popular social networking software in China (17), users can communicate with other users individually or establish a group to communicate together through WeChat. In this study, The WeChat group chat was managed by the program team, and each community established a group chat with members including survey respondents, as well as general practitioner team members, smoking cessation clinic physicians, program team members, and a graduate student majoring in psychology.

### 2.3. Interventions

In this study, communities were divided into pilot and control groups with matched socio-economic, population health and community health service characteristics and smokers were allocated to either the pilot or control group depending on their community. A double-blind approach was used throughout the intervention. In the control group, a 5A brief smoking cessation intervention (18) was implemented. After being asked about tobacco use and related health information at the baseline, smokers were given personalized guidance and advice on their smoking cessation plan. If smokers needed the assistance of smoking cessation medication, physician from smoking cessation clinic would decide whether to provide medication based on the smoker’s medical condition and medication use, etc. At the same time, smokers were informed of other ways to obtain smoking cessation support, such as visiting hospital’s smoking cessation clinic, calling the smoking cessation hotline. After that, no additional interventions are made except for a brief intervention for the control group at the follow-up.

In addition to brief smoking cessation interventions, the interventions in the pilot group also included online and offline health education activities. Interventions in the pilot and control groups are shown in Table 1. The offline activities were mainly held in community health centers, where smoking cessation clinicians provided medication and counseling guidance to quitters. Special thematic educational activities would also be held, such as lectures on smoking cessation knowledge, peer group activities on smoking cessation, etc. On average, each community in the pilot group offered 1–2 times during the intervention period. The online interventions were mainly implemented through the smoking cessation WeChat groups in each community. Interventions include:

**TABLE 1:** Interventions in the pilot and control groups.

	Baseline	1st month	3rd month	6th month
Control group	Questionnaire brief smoking cessation intervention	Brief smoking cessation intervention follow-up	Brief smoking cessation intervention follow-up	Brief smoking cessation intervention follow-up
Pilot group	Questionnaire brief smoking cessation intervention online activities offline activities	Brief smoking cessation intervention online activities offline activities follow-up	Brief smoking cessation intervention online activities offline activities follow-up	Brief smoking cessation intervention follow-up

(a) Organizing health education professionals to regularly send out weekly educational materials.

(b) Providing online answers to questions and concerns by smoking cessation clinics and general practitioners.

(c) Providing psychological coping plans for quitters with withdrawal symptoms by psychological professionals.

(d) Notifying activities and follow-up, online discussions among group members, and sharing feelings and experiences about quitting smoking.

(e) Providing online professional counseling and guidance for psychological barriers brought on by withdrawal symptoms. At the same time, the program team designed and produced promotional materials such as smoking cessation-related folders, posters, and easy-to-use posters for tobacco-dependent patients at different stages. No further online and offline interventions were conducted in the pilot group after 3 months’ intervention.

After conducting the intervention, follow-up was conducted at week 4 (1st month), week 12 (3rd month), and week 24 (6th month). General practitioner in community health institutes and doctors in smoking cessation clinicians of general hospital were involved in the follow-up conducted at community health center. At the follow-up, smoking cessation status of quitters was recorded along with the number of cigarettes smoked in the week before the follow-up. Quitters who were present had the CO testing, and those who were absent were followed up by telephone and their smoking cessation medications were delivered to them by the general practitioners.

### 2.4. Outcomes

Average number of cigarettes smoked per day (ACSD) at the 1st, 3rd, and 6th month and smoking cessation rates in the 3rd and 6th month were selected as the evaluation index of the intervention effect. There is no standardized criterion for successful smoking cessation, 7-day point prevalence abstinence and 30-day point prevalence abstinence, as well as long- term/prolonged/sustained rates abstinence to evaluate the effectiveness of smoking cessation interventions (19–21). This paper defines successful smoking cessation as a smoker’s self-reported ACSD of 0 at the follow-up. In addition, the integrated intervention in this study was stopped at 3rd month. Intention-to-treat analysis (ITT analysis) (12, 22) method was used for those who fell off midway through the study. If smokers did not attend the follow-up, their ACSD at the most recent follow-up was used as the outcome of the current follow-up, as was their smoking cessation status. Related studies have shown that using ITT followed by analysis leads to conservative results (23, 24).

### 2.5. Analysis

Statistical Product and Service Solutions 19.0 (SPSS 19.0) was used to process the data. Descriptive statistics were used to report demographic information of the population in the pilot and control groups. Differences in demographic information and smoking cessation rates between the two groups were analyzed using chi-square tests. Measures were expressed as mean ± standard deviation ($\hat{x}\pm s$). The effects of the intervention and medications taking on ACSD were analyzed using generalized estimating equations (25). Differences were considered statistically significant at a two-sided *P* ≤ 0.05.

The generalized estimating equations (GEE) were used instead of traditional regression to explore the association between factors (26) or to predict the influencing factors. Its main feature is the introduction of the working correlation matrix, which takes into consideration the correlation between the outcome variables of repeated measures information (27). The form of the working correlation matrix should be predetermined before fitting the model. Related studies have pointed out that the choice of the working correlation matrix has little effect on parameter estimation (28). In the present study, which was the same cohort population from intervention to follow-up, the ACSD may be correlated, so an unstructured working correlation matrix was chosen for the analysis.

In the analysis of repeated data such as GEE, if there is an interaction effect, then the difference between the main effect and the difference between the overall mean at the corresponding level does not correspond. A further simple effects analysis should be performed to infer whether there is a difference between the corresponding means of a variable at different levels of another variable (29).

## 3. Result

**FIGURE 1:**
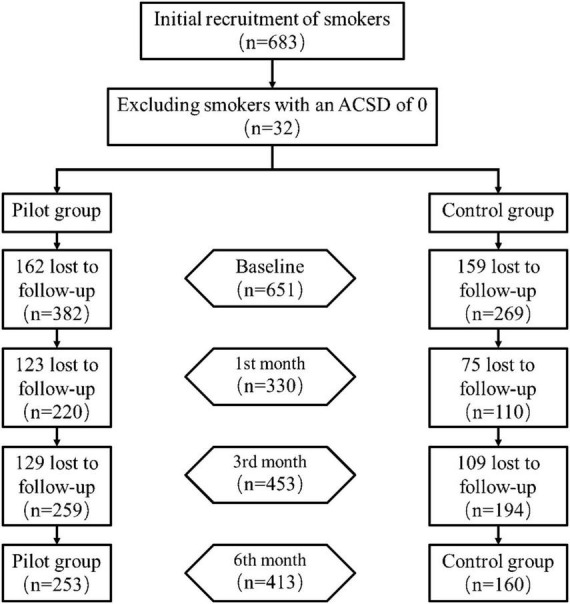
Baseline and follow-up of smokers included in the study.

A total of 683 smokers with the intention to quit were recruited in this study. Of these, 32 reported an ACSD of 0 at baseline survey and were not included in this study. Of the remaining 651, 382 were included in the pilot group and 269 in the control group, and the follow-up is shown in Figure 1. The largest number of people were lost at the first follow-up (321, 49.3%), and 413 (63.4%) were not shed at the end of the intervention.

The demographic characteristics of smokers in both groups are shown in Table 2. The main characteristics of the population in this survey were middle-income, urban household, middle-aged, and married males. The chi-square test showed no significant differences in distribution of the pilot and control groups in terms of gender, monthly income level, and average daily smoking (*P* > 0.05). There was a significant difference in distribution of smokers in terms of age, marital status, education, work type, registered residence, and health insurance (*P* < 0.05).

**TABLE 2:** The demographic characteristics of smokers in study (*N* = 651, *P < 0.05).

Demographic characteristics		Total	Pilot group (*n* = 382)		Control group (*n* = 269)	
			**Frequency**	**Percent (%)**	**Frequency**	**Percent (%)**
**Gender**						
Male		590	352	92.1	238	88.5
Female		61	30	7.9	31	11.5
Age*						
Under 39 years old		100	49	12.9	51	19.0
40–49 years old		98	49	12.9	49	18.3
50–59 years old		192	112	29.5	80	29.9
60 years old and above		258	170	44.7	88	32.8
Marriage*						
Married		568	342	90.2	226	84.3
Others		79	37	9.8	42	15.7
**Average monthly income (RMB)**						
Below 2,000		71	40	11.7	31	13.2
2,001–4,000		187	111	32.4	76	32.5
4,001–6,000		152	103	30.0	49	20.9
6,001–8,000		63	34	9.9	29	12.4
8,001–10,000		45	23	6.7	22	9.4
10,001 and more		59	32	9.3	27	11.5
Education*						
Elementary school and below		44	16	4.2	28	10.4
Middle and high school		345	208	54.7	137	51.1
College and above		259	156	41.1	103	38.4
Work type*						
Production staff, operators, or clerical staff		67	37	9.8	30	11.3
Commercial or service industry personnel		76	33	8.8	43	16.2
State organs, enterprises, and institutions personnel		68	43	11.4	25	9.4
Professional and technical staff		57	29	7.7	28	10.5
Military, students, unemployed, and other workers		96	49	13.0	47	17.7
Retirees		279	186	49.3	93	35.0
Registered residence*						
Urban		528	326	87.6	202	78.3
Rural		102	46	12.4	56	21.7
Health insurance*						
Urban employee insurance		377	241	63.6	136	51.1
Urban residents’ insurance		154	90	23.7	64	24.1
Others		114	48	12.7	66	24.8
**ACSD (Average cigarettes smoked per day)**						
		651	17.75 ± 9.509		17.17 ± 9.805	

### 3.1. Analysis of generalized estimating equations of ACSD

The model effect test results generalized estimating equation showed that there was a main effect of time, medication taking, and intervention factors (*P* < 0.05), as well as an interaction effect of those three factors (*P* < 0.05). When there is an interaction effect, the equation analysis is mainly based on the results of simple effect analysis.

The results of simple effects analysis of medication taking factor showed that at baseline, there was no significant difference in ACSD in smokers taking the medication compared to smokers not taking the medication in either the control or pilot group (*P*-values of 0.703 and 0.526, respectively, both greater than 0.05). After 1, 3, and 6 months of intervention, there was a significant difference in ACSD in smokers taking medication compared to those not taking medication (*P* < 0.05). In terms of mean differences, at each follow-up, smokers taking medication had lower ACSD than those not taking medication, and the difference decreased over time, as detailed in Table 3.

**TABLE 3:** Simple effect analysis of medication taking factors.

Group	Time	Mean difference*	SE	*P*
Control group	Baseline	−0.473 (−2.902, 1.956)	1.239	0.703
	1st month	3.270 (0.707, 5.829)	1.307	0.012
	3rd month	4.830 (2.445, 7.224)	1.219	*P* < 0.001
	6th month	4.760 (2.217, 7.307)	1.299	*P* < 0.001
Pilot group	Baseline	−0.669 (−2.736, 1.399)	1.055	0.526
	1st month	6.230 (4.233, 8.233)	1.020	*P* < 0.001
	3rd month	5.820 (3.949, 7.684)	0.953	*P* < 0.001
	6th month	4.100 (2.176, 6.033)	0.984	*P* < 0.001

*Mean difference between ACSD of Non-medication-taking smokers and medication-taking smokers.

The results of time factor simple effects analysis for non-medication-taking group showed that there was a significant difference in ACSD between the pilot and control groups at each follow-up (*P* < 0.05). The mean difference showed that ACSD among smokers decreased significantly with the intervention time. The results of time factor simple effects analysis for medication-taking group showed that there was a significant difference (*P* < 0.05) in ACSD among smokers in the control group at each follow-up, with a significant reduction of 8.650 cigarettes per day in 6 months of intervention. In addition, the difference between ACSD at the two adjacent follow-up decreased gradually, the reduction in ACSD mainly occurring in the first 3 months after the intervention. The ACSD of smokers in the pilot group at each follow-up after the intervention was significantly different from the ACSD at baseline (*P* < 0.05), the ACSD after 1 month of the intervention was significantly different from the ACSD after 3 and 6 months of the intervention (*P* < 0.05), and the ACSD after 3 months of the intervention was not significantly different from the ACSD after 6 months of the intervention (*P* = 0.708), as detailed in Table 4.

**TABLE 4:** Simple effect analysis of time factor.

	Group	Time (I)	Time (J)	Mean difference (I-J)	SE	*P*
Non-medication-taking	Control group	Baseline	1st month	1.120 (0.351, 1.884)	0.391	0.004
		Baseline	3rd month	2.390 (1.011, 3.766)	0.703	0.001
		Baseline	6th month	3.410 (1.832, 4.992)	0.806	*P* < 0.001
		1st month	3rd month	1.270 (0.004, 2.537)	0.646	0.049
		1st month	6th month	2.290 (0.786, 3.803)	0.770	0.003
		3rd month	6th month	1.020 (0.113, 1.934)	0.465	0.028
	Pilot group	Baseline	1st month	0.890 (0.242, 1.540)	0.331	0.007
		Baseline	3rd month	2.78 (1.406, 4.145)	0.699	*P* < 0.001
		Baseline	6th month	4.600 (2.879, 6.323)	0.879	*P* < 0.001
		1st month	3rd month	1.880 (0.684, 3.085)	0.612	0.002
		1st month	6th month	3.710 (2.086, 5.335)	0.829	*P* < 0.001
		3rd month	6th month	1.830 (0.658, 2.994)	0.596	0.002
Medication-taking	Control group	Baseline	1st month	4.860 (3.798, 5.919)	0.541	*P* < 0.001
		Baseline	3rd month	7.700 (6.444, 8.948)	0.639	*P* < 0.001
		Baseline	6th month	8.650 (7.334, 9.959)	0.670	*P* < 0.001
		1st month	3rd month	2.840 (1.794, 3.880)	0.532	*P* < 0.001
		1st month	6th month	3.790 (2.606, 4.970)	0.603	*P* < 0.001
		3rd month	6th month	0.950 (0.184, 1.718)	0.391	0.015
	Pilot group	Baseline	1st month	7.790 (6.758, 8.828)	0.528	*P* < 0.001
		Baseline	3rd month	9.260 (8.057, 10.463)	0.614	*P* < 0.001
		Baseline	6th month	9.380 (8.124, 10.626)	0.638	*P* < 0.001
		1st month	3rd month	1.470 (0.546, 2.388)	0.470	0.002
		1st month	6th month	1.580 (0.564, 2.600)	0.519	0.002
		3rd month	6th month	0.115 (−0.485, 0.714)	0.306	0.708

Results of intervention factor simple effects analysis for non-medication taking smokers showed that there was no significant difference in ACSD between the pilot and control groups at each follow up (*P* < 0.05). Results of the intervention factor simple effect analysis for medication taking smokers showed that there was no significant difference in ACSD between pilot and control groups at baseline (*P* > 0.05). Compared to the control group, the ACSD in pilot group decreased by 3.420 and 2.050 cigarettes after 1 and 3 months of intervention, both of which were significantly different (*P* < 0.05). After 6 months of intervention, there was no significant difference between this two groups in terms of ACSD (*P* = 0.141). The effect of integrated intervention was mainly concentrated in first 3 months, as detailed in Table 5.

**TABLE 5:** Simple effect analysis of intervention factor.

	Time	Mean difference*	SE	*P*
Non-medication-taking	Baseline	0.680 (−1.930, 3.300)	1.332	0.607
	1st month	0.460 (−2.220, 3.130)	1.365	0.737
	3rd month	1.070 (−1.520, 3.670)	1.324	0.418
	6th month	1.870 (−0.880, 4.630)	1.404	0.182
Medication-taking	Baseline	0.489 (−1.343, 2.320)	0.934	0.601
	1st month	3.420 (1.580, 5.266)	0.940	*P* < 0.001
	3rd month	2.050 (0.484, 3.623)	0.801	0.010
	6th month	1.217 (−0.402, 2.837)	0.826	0.141

*Mean difference between ACSD of control group and pilot group.

**FIGURE 2:**
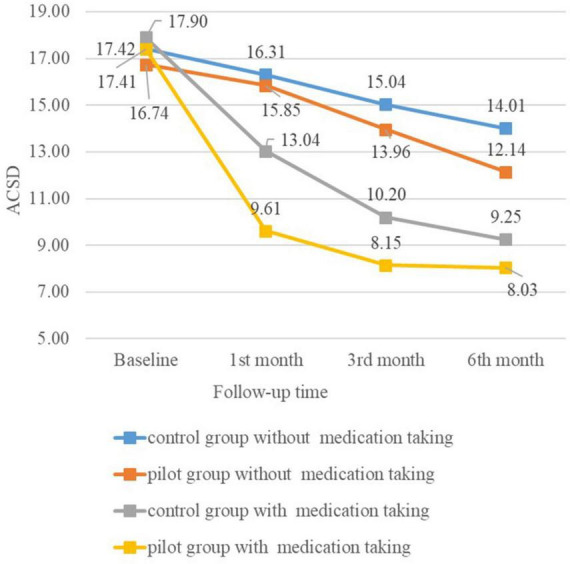
Mean marginal estimates by medication-taking subgroup.

Figure 2 shows the mean marginal estimates of ACSD in the control group and the pilot group at each follow-up after grouping according to whether they took medication.

### 3.2. Chi-square test of smoking cessation rates of smokers

Grouped according to whether they were taking medication or not, the smoking cessation rates were calculated separately for the control and pilot groups after 3 and 6 months’ intervention, and the results are shown in Table 6. The difference in smoking cessation rates of non-medication taking smokers between the pilot and control groups was not significant (*P* > 0.05), implying that the brief and integrated interventions were similar in promoting smoking cessation among non-medication smokers. There was a significant difference in smoking cessation rates of medication taking smokers between the pilot and control groups at 3rd month (*P* = 0.019). However, after stopping the intervention, the difference in smoking cessation rates between the two groups at the 6th month was not significant (*P* = 0.231).

**TABLE 6:** Analysis of chi-square test for smoking cessation rates at 3rd month and 6th month.

	Group	3rd month		6th month	
		**Number of quitters**	**Percentage (%)**	**Number of quitters**	**Percentage (%)**
Non-medication-taking	Control group	6	7.1	13	15.3
	Pilot group	10	7.2	22	15.9
*χ^2^, p*		0.003, 0.958		0.017, 0.897	
Medication-taking	Control group	32	17.4	44	23.9
	Pilot group	66	27.0	71	29.1
*χ^2^, p*		5.542, 0.019		1.435, 0.231	

## 4. Discussion

**FIGURE 3:**
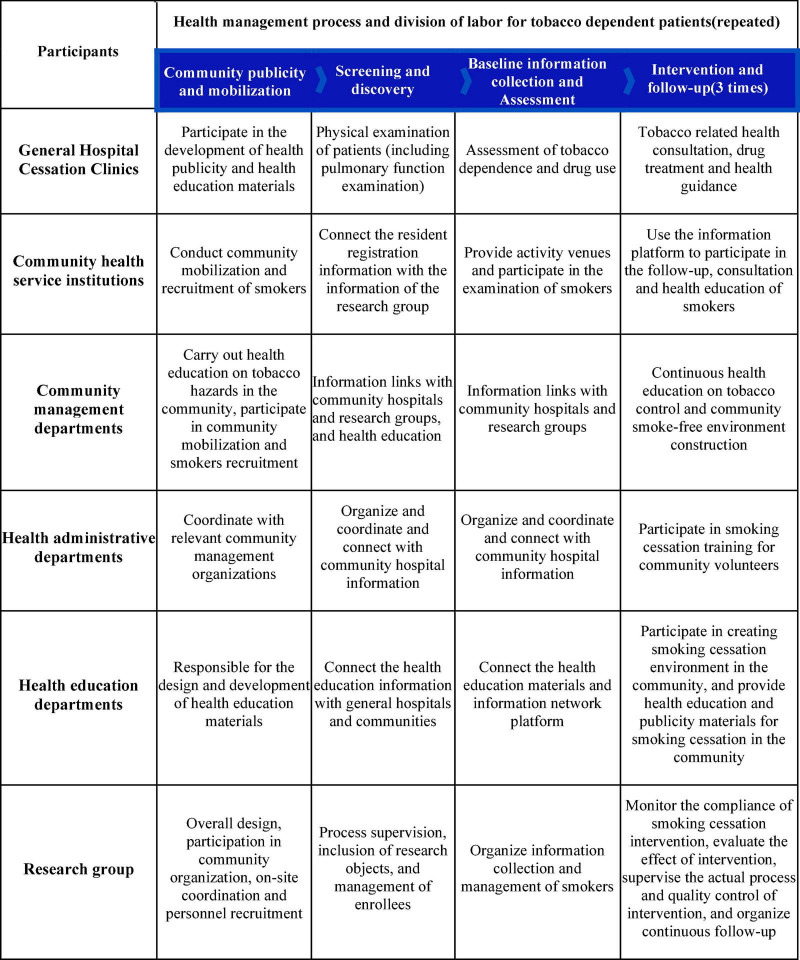
Management pattern of tobacco dependence in hospital and community.

This study combined a general hospital and a community health centers, taking advantage of the technical strengths of the smoking cessation clinics in general hospital and the distance advantages of the community health centers. The general hospital trained community doctors in smoking cessation skills, and the community smoking cessation clinic conducted follow-up assessments of patients. After the implementation of the general practitioner contracting system in China, the relationship between general practitioners and their contracted families is more stable and communication between doctors and patients is relatively smooth, which is helpful for timely health guidance (30, 31). Currently, community health workers have more positive attitudes toward tobacco control, but their behavior in delivering smoking cessation interventions is less than ideal (32), providing a favorable opportunity for programme implementation in the community. Therefore, with the concerted efforts of these staff, a better operation mode have been formed, in which the health administration department and social management department provided support, and the research group organized and implemented systematic hospital community smoking cessation intervention, see Figure 3. Studies in the United States (33), the United Kingdom (13), and Thailand (14) have shown that community-based interventions have good effects on smoking cessation, and these studies demonstrate the critical importance of the external environment in helping smokers to quit (34). In this study, several social organizations and institutions were included in the design of the intervention. For example, the Beijing Association for Tobacco Control assisted in completing social mobilization and recruitment of patients; the China Health Education Center produced smoking cessation publicity materials; and community committees also contributed to the recruitment of smokers and follow-up visits, building a good external support environment for smokers.

First, this study verified the effectiveness of smoking cessation medications for smoking cessation. In both the pilot and control groups, smokers taking the medication had a significantly lower ACSD at each follow-up than smokers not taking medication, and smoking cessation rates were higher than those who did not take medication. Second, this study demonstrated the effectiveness of the brief intervention for smoking cessation. The ACSD at the follow-up was significantly lower than that at the previous follow-up in all groups, except for the test group where the difference between the ACSD at 6th month and 3rd month was not significant. This is because both the pilot and control groups included brief smoking cessation interventions, and various previous studies have demonstrated that brief smoking cessation interventions can be effective in increasing smoking cessation rate (35, 36), with the 6th month smoking cessation rate in the pilot group with medication taking in this study being higher than the 21.6% smoking cessation rate in Lin et al. (35). Finally, this study demonstrates that an integrated intervention can effectively reduce ACSD and increase smoking cessation rate in smokers taking smoking cessation medications. After 1 and 3 months of the intervention, smokers in the pilot group with medication taking had significantly lower ACSD than smokers in the control group with medication taking, had significantly lower smoking cessation rate at 3rd month, and had non-significant differences in ACSD and smoking cessation rate at 6th month between the two groups after stopping the intervention. While the differences in ACSD and smoking cessation rates between smokers in the pilot and control groups without medication taking were consistently non-significant at each follow-up.

Related studies have shown that supplementation with medication has better results than intervention alone for smokers who do not want to quit (37). In the present study, there was no difference in the effect of brief and integrated smoking cessation interventions on ACSD in smokers who did not take medication to quit. In contrast, the integrated intervention was more effective when medication was taken. This seems to suggest that the use of smoking cessation medication is superior to either the brief smoking cessation intervention or the integrated smoking cessation intervention in smoking cessation intervention. Compared to the brief intervention, taking medication means that smokers have to pay for the medication. The cost of smoking cessation medications in this study was covered by program funding and was provided free of charge to smokers. Although smoking cessation medications are cost effective (38) and some studies have shown that the average expenditure on smoking cessation medications for smokers covered by Medicaid is $0.15 per month (39). However, in a study of smoking cessation clinics in China, up to 62% of smokers attending the clinic did not consider smoking as a disease (40), and they were prone to the perception that they did not need medication to quit smoking. Moreover, smoking cessation medications in China are not included in the health insurance reimbursement list, and the cost of paying for medications may not be lower than the cost of purchasing cigarettes, while medications may also have side effects. These phenomena may lead smokers not to choose smoking cessation medication, which suggests that we should strengthen the publicity on the dangers of smoking and related knowledge, and include smoking cessation medication in the medical insurance reimbursement catalog as soon as possible to reduce the financial burden of smokers.

In addition, community-based smoking cessation interventions increase contact with smokers and to some extent improve adherence. Medication adherence can significantly affect the effectiveness of smoking cessation (41), and a lack of awareness of smoking cessation pharmacotherapy, a perception that smoking cessation medications are ineffective, or side effects of taking medications can lead to lower adherence among smokers (42). In this study, smokers were able to exchange their quit experiences in a WeChat group or consult with professionals, and most of the problems of low medication adherence could be solved through this way. This not only eliminated smokers’ concerns, but also increased their confidence in quitting. This format increases the smoker’s access to the interventionist and increases the intensity of the intervention, which in turn increases the smoker’s adherence to quit. Some studies have shown low adherence among smokers in population-based cessation interventions (43), which may be due to the weak intensity of the intervention (34). This suggests that when designing community-based interventions, the focus should be on the intensity of the intervention and how to improve smokers’ adherence.

There are some limitations in this study. In terms of statistical analysis, firstly, smokers’ adherence was not evaluated and recorded in this study. The loss of smokers to follow up in the first month in the control group resulted in a control group with fewer than the minimum number of people required for the study (245) and may have contributed to bias in the analysis. This may also be a side-effect of the role of integrated interventions in enhancing smokers’ compliance with the intervention. Second, the generalized estimating equation used in this study included only three independent variables for analysis, namely time, group and whether medication was used, and did not control for variables such as age and marriage, which had significantly different distributions in the groups, which may have had some effect on the results of the analysis. In the promotion of interventions, firstly, the intervention forms such as WeChat group chat are additional services provided by physicians, and most responses can only be made during non-working hours. If this integrated intervention is popularized, the physicians involved in the intervention will not only increase their workload, but also receive no additional remuneration, which will affect their enthusiasm over time. The same problem was encountered in the UK study (13). Secondly, the issue of payment for smoking cessation medications suggests that how to quantify and pay doctors for their extra work, and how to charge smokers for medical behaviors in informal organizations such as WeChat, is an issue that needs to be addressed in popularizing this integrated intervention model. Finally, the design and implementation of this study is mainly based on the good social support environment for tobacco control in Beijing, which is not available in most other provinces in China, which also brings limitations to the promotion of this study conclusion.

## 5. Conclusion

An integrated hospital-community tobacco dependence management model can provide a good external supportive environment for smokers and is effective in increasing adherence and reducing ACSD. However, the prerequisite is the need to take smoking cessation medication. In addition, the social organizations involved in the study and the additional services generated by the medical staff need to be further studied through economic evaluation to ensure that the model is cost-effective and worthy of being popularized to other areas.

## Data availability statement

The raw data supporting the conclusions of this article will be made available by the authors, without undue reservation.

## Ethics statement

The studies involving human participants were reviewed and approved by the Medical Ethics Committee of Capital Medical University (Z2019SY007) and has been registered in the China Clinical Trials Registry under the name Search for Optimization of Tobacco Dependence Management Model Based on Hospital and Community (ChiCTR1900024991). The patients/participants provided their written informed consent to participate in this study.

## Author contributions

KQ: conceptualization, methodology, data analysis, writing–original draft, and writing–review and editing. HL: conceptualization, data collection, and analysis. XL: conceptualization, methodology, investigation, resources, supervision, project administration, and writing–review and editing. QJ: investigation, project management, and data collection. YW: investigation, resources, and project management. MG: investigation and data and project management. XB: writing–review and editing. TQ: conceptualization, investigation, and data management. YY: resources and projects. All authors contributed to the article and approved the submitted version.
